# Intrinsic *Gata4* expression sensitizes the aortic root to dilation in a Loeys-Dietz syndrome mouse model

**DOI:** 10.21203/rs.3.rs-4420617/v1

**Published:** 2024-06-05

**Authors:** Emily E. Bramel, Wendy A. Espinoza Camejo, Tyler J. Creamer, Leda Restrepo, Muzna Saqib, Rustam Bagirzadeh, Anthony Zeng, Jacob T. Mitchell, Genevieve L. Stein-O’Brien, Albert J. Pedroza, Michael P. Fischbein, Harry C. Dietz, Elena Gallo MacFarlane

**Affiliations:** 1 McKusick-Nathans Department of Genetic Medicine, Johns Hopkins University School of Medicine, Baltimore, Maryland, USA; 2 Predoctoral Training in Human Genetics and Genomics, Johns Hopkins University School of Medicine, Baltimore, Maryland, USA; 3 Department of Surgery, Johns Hopkins University School of Medicine, Baltimore, Maryland, USA; 4 Solomon H. Snyder Department of Neuroscience, Johns Hopkins University School of Medicine, Baltimore, Maryland, USA; 5 Department of Cardiothoracic Surgery, Stanford University School of Medicine, Stanford, California, USA

## Abstract

Loeys-Dietz syndrome (LDS) is an aneurysm disorder caused by mutations that decrease transforming growth factor-β (TGF-β) signaling. Although aneurysms develop throughout the arterial tree, the aortic root is a site of heightened risk. To identify molecular determinants of this vulnerability, we investigated the heterogeneity of vascular smooth muscle cells (VSMCs) in the aorta of *Tgfbr1*^*M318R*/+^ LDS mice by single cell and spatial transcriptomics. Reduced expression of components of the extracellular matrix-receptor apparatus and upregulation of stress and inflammatory pathways were observed in all LDS VSMCs. However, regardless of genotype, a subset of *Gata4*-expressing VSMCs predominantly located in the aortic root intrinsically displayed a less differentiated, proinflammatory profile. A similar population was also identified among aortic VSMCs in a human scRNAseq dataset. Postnatal VSMC-specific *Gata4* deletion reduced aortic root dilation in LDS mice, suggesting that this factor sensitizes the aortic root to the effects of impaired TGF-β signaling.

Thoracic aortic aneurysms are localized vascular dilations that increase the risk of fatal dissections and/or rupture of the vessel wall^1^. Effective medical therapies to prevent life-threatening aortic events remain elusive^2^. Loeys-Dietz syndrome (LDS) is a hereditary connective tissue disorder that presents with highly penetrant aortic aneurysms^3,4^. LDS is caused by heterozygous, loss-of-function mutations in positive effectors of the TGF-β signaling pathway, including receptors (*TGFBR1, TGFBR2)*, ligands (*TGFB2, TGFB3*) and intracellular signaling mediators (*SMAD2, SMAD3*)^5–9^. All of these mutations result in reduced phosphorylation/activation of Smad2 and Smad3, leading to defective Smad-dependent transcriptional regulation. Secondary compensatory mechanisms, including upregulation of Angiotensin II Type I Receptor (AT1R) signaling, and increased expression of TGF-β ligands and Smad proteins, ultimately elevate levels of Smad2/Smad3 activity at diseased aortic sites, with outcomes ranging from adaptive to maladaptive depending on disease progression and cellular context^5,7,10–13^. While LDS-causing mutations heighten aneurysm risk in all arteries, the aortic root is especially vulnerable to disease^14–17^. Several laboratories have highlighted how the cellular composition and/or the mechanical stresses may contribute to the increased risk of disease in this location, however, the molecular determinants of this susceptibility remain unclear^13,18–22^. Additionally, VSMCs are the primary cellular component of the aortic wall, but the heterogeneity of VSMCs within the aorta and its implications for aneurysm are not fully understood. In this study, we investigate the transcriptional heterogeneity of VSMCs in the normal and diseased murine aorta leveraging both scRNAseq and spatial transcriptomics. We identify *Gata4* as a regional factor whose expression is intrinsically elevated in the aortic root and further upregulated in LDS samples. We also show that postnatal deletion of *Gata4* in VSMCs ameliorates aortic root dilation in a murine model of LDS harboring a *Tgfbr1*^*M318R*/+^ genotype.

## Results

### *Tgfbr1*^*M318R*/+^ VSMCs downregulate extracellular matrix components, focal adhesions, and integrin receptors, and upregulate transcripts related to stress and inflammatory pathways.

**Figure 1.**
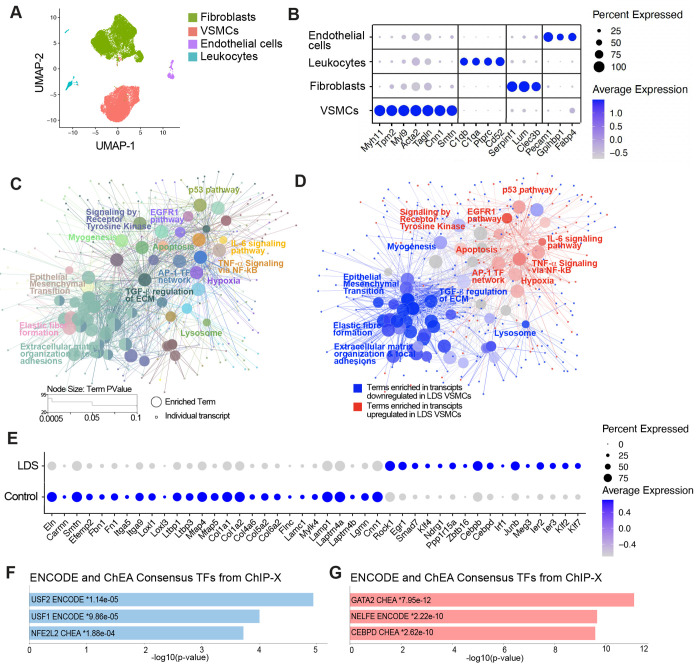
Downregulation of transcripts associated with extracellular matrix-receptor interactions and upregulation of stress and inflammation pathways in *Tgfbr1*^*M318R*/+^ LDS VSMCs. (A) Uniform manifold approximation and projection (UMAP) of aortic cells from control (*Tgfbr1*^+/+^) and LDS (*Tgfbr1*^*M318R*/+^) mice. (B) Dot plot of cluster defining transcripts used to identify endothelial cells, leukocytes, fibroblasts, and VSMCs. Color of the dot represents a scaled average expression while the size indicates the percentage of cells in which the transcript was detected. (C) ClueGO gene enrichment analysis network of transcripts dysregulated in LDS VSMCs relative to controls. Each node represents a term/pathway or individual genes associated with that term. The color of the node corresponds to the ClueGO group to which each node belongs. The size of the node indicates significance of the enrichment calculated by the ClueGO algorithm. (D) ClueGO network in which terms differentially enriched among transcripts downregulated in LDS VSMCs are highlighted in blue, while those enriched among transcripts upregulated in LDS VSMCs are highlighted in red. (E) Dot plot showing expression of a selection of transcripts significantly dysregulated in LDS VSMCs. (F,G) EnrichR gene over-representation analysis for the ENCODE and ChEA Consensus transcription factors (TF) databases showing the top three most significant terms associated with transcripts that are downregulated (F) or upregulated (G) in LDS VSMCs.

LDS mouse models expressing a heterozygous missense mutation in *Tgfbr1* (*Tgfbr1*^*M318R*/+^) develop highly penetrant aortic root aneurysms^11,13^. To assess transcriptomic changes associated with vascular pathology in this model, we performed single cell RNA sequencing (scRNAseq) on the aortic root and ascending aorta of control (*Tgfbr1*^+/+^) and LDS mice at 16 weeks of age, resulting in the identification of all of the expected cell types according to well-established expression profiles^23^ (Fig. 1A, B and Supplemental Fig. 1). In consideration of the critical role of VSMCs in the pathogenesis of aortic aneurysm^24,25^, we focused the downstream analysis of LDS-driven transcriptional alterations on this cell type (Supplemental Table 1). Using the Cytoscape^26^ ClueGO^27^ plug-in to leverage gene set enrichment information from multiple databases, we produced a network of functionally related terms and pathways that are differentially enriched among downregulated and upregulated transcripts. (Fig. 1C, D and Supplemental Table 2). The *Tgfbr1*^*M318R*/+^ LDS mutation caused broad downregulation of transcripts related to the maintenance of extracellular matrix-receptor interactions, and integrity of the elastic and contractile function of the aortic wall (Fig. 1C, D, E and Supplemental Table 2). Concurrently, pathways involved in cellular stress responses, inflammation, senescence, and cell death were enriched among transcripts upregulated in *Tgfbr1*^*M318R*/+^ VSMCs (Fig. 1C, D, E and Supplemental Table 2). Additional analysis of transcription factor target databases (ENCODE^28^ and Chromatin Immunoprecipitation Enrichment Analysis (ChEA) via EnrichR^29–32^) showed that LDS-downregulated transcripts were enriched in targets of NFE2L2 (nuclear factor erythroid 2-related factor 2, also known as Nrf2), a transcription factor that activates expression of cytoprotective genes and suppresses expression of proinflammatory mediators^33–35^ (Fig. 1F and Supplemental Table 2). Targets of the upstream stimulatory factor (USF) family, which can modulate the expression of smooth muscle specific genes were also enriched among downregulated transcripts^36–39^ (Fig. 1F and Supplemental Table 2). Conversely, target genes for GATA transcription factors and CCAAT enhancer binding protein delta (CEBPD), a positive transcriptional regulator of inflammatory responses mediated by interleukin-1 (IL-1) and IL-6^40–43^, were enriched among transcripts upregulated in LDS VSMCs (Fig. 1G and Supplemental Table 2).

### Spatial transcriptomic analysis of the murine aorta reveals region- and disease-specific patterns of expression for modulators of VSMC phenotypes.

**Figure 2.**
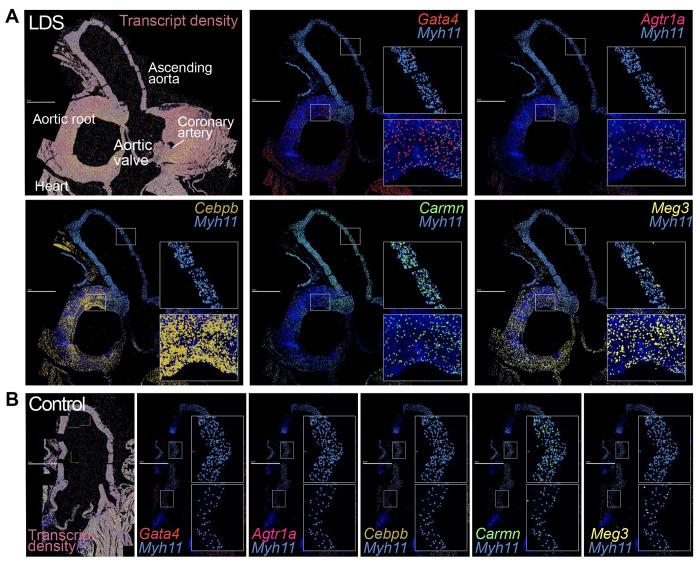
MERFISH reveals spatially heterogeneous transcriptional profiles in LDS VSMCs. MERFISH images of the proximal aorta of LDS (A) and control (B) mice, scale bar is 1 mm. The first panel displays all detected transcripts across the aortic tissue, with key anatomic landmarks indicated. Subsequent panels depict the colocalization of *Myh11* and transcripts of interest. Insets note regions of the ascending aorta and aortic root that are presented at higher magnification.

Given the regional vulnerability observed in LDS aortas, we leveraged insight gained from the literature and scRNAseq analysis of the aorta of control and *Tgfbr1*^*M318R*/+^ mice to design a custom panel for high throughput in situ hybridization using the Multiplexed error-robust fluorescence in situ hybridization (MERFISH) spatial transcriptomics platform (Supplemental Table 3). Analysis of a longitudinal section of the proximal aorta of 16-week-old control and LDS mice showed regionally defined expression of several transcripts involved in the modulation of vascular phenotypes (Fig. 2 and Supplemental Fig. 2). Transcripts more highly detected in the aortic root of LDS mice relative to the ascending aorta included *Agtr1a*, which codes for angiotensin II receptor type 1a, a known contributor to LDS pathogenesis, and *Gata4*, which codes for a transcription factor known to positively regulate *Agtr1a* expression in the heart^44,45^. CCAAT enhancer binding protein beta (*Cebpb*), a pro-inflammatory mediator^46^, and maternally expressed gene 3 (*Meg3*), a long non-coding RNA (lncRNA) that negatively regulates TGF-β signaling and promotes VSMC proliferation^47–50^, were also enriched in this region. In contrast, expression of cardiac mesoderm enhancer-associated noncoding RNA (*Carmn*), a positive regulator of VSMC contractile function that is downregulated in vascular disease, and expression of *Myh11*, a marker of differentiated VSMCs, was enriched in the distal ascending aorta, a region that is only mildly affected in LDS mouse models^49,51–53^.

### Expression of cluster-defining transcripts for the VSMC2 and VSMC1 subclusters correlates with the proximal-to-distal axis of the mouse and human aorta.

**Figure 3.**
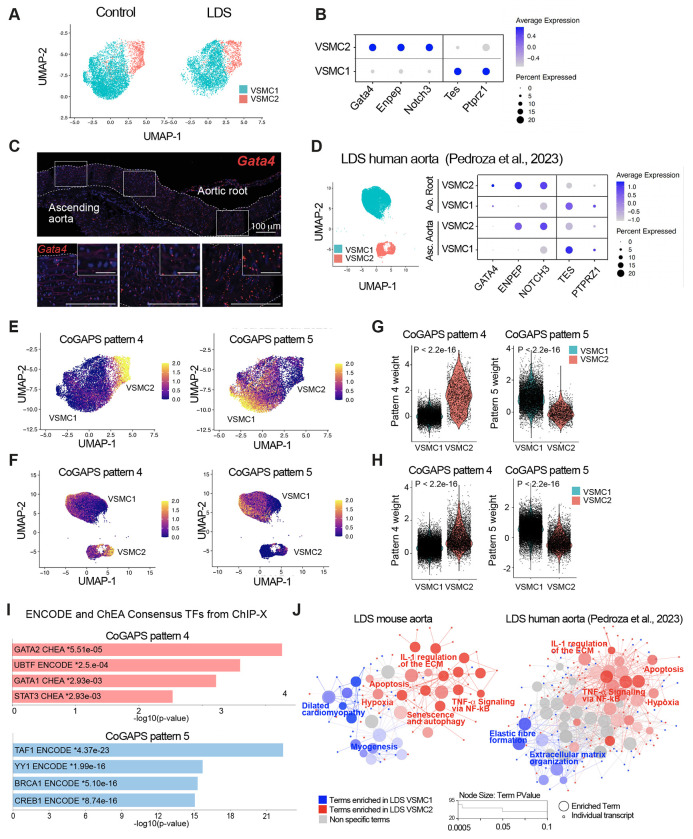
Transcriptionally and spatially-defined VSMC subclusters with distinct responses to LDS-causing mutations can be identified in both murine and human aortas. (A) UMAP of VSMCs from control (*Tgfbr1*^+/+^) and LDS (*Tgfbr1*^*M318R*/+^) mice shown split by genotype. (B) Dot plot showing enrichment of cluster-defining transcripts in VSMC1 and VSMC2. For a given transcript, the color of the dot represents a scaled average expression while the size indicates the percentage of cells in which it was detected. (C) RNA in situ hybridization showing the expression of *Gata4* along the length of the murine aorta in a 16-week old control animal. (D) UMAP of control and LDS VSMCs from human patients and dot plot of cluster defining markers in this dataset split by aortic region (Pedroza et al., 2023). (E,F) UMAP overlayed with weights for CoGAPS patterns 4 and 5, in mouse and human scRNAseq datasets. (G,H) Violin plots showing the distribution of pattern 4 and 5 weights in VSMC subclusters from mouse and human scRNAseq datasets. P-values refer to Wilcoxon test. (I) EnrichR gene over-representation analysis for the ENCODE and ChEA Consensus TF databases showing the top four most significant terms associated with transcripts that define CoGAPs Patterns 4 and 5. (J) ClueGO network of terms differentially enriched in mouse and human LDS VSMC2 relative to VSMC1. Terms highlighted in blue are enriched in VSMC1, while those highlighted in red are enriched in VSMC2.

To examine if the spatial VSMC heterogeneity observed with MERFISH could be captured by scRNAseq, we increased the clustering resolution for VSMCs, thus obtaining two subclusters, VSMC1 and VSMC2. We then examined these two VSMC subclusters for expression of transcripts our laboratory has previously shown to progressively increase (i.e. *Tes* and *Ptprz1*) and decrease (i.e. *Enpep* and *Notch3*) along the proximal-to-distal axis in the mouse ascending aorta^54^. VSMC1 and VSMC2 showed increased expression of transcripts whose expression is intrinsically enriched in the ascending aorta and the aortic root, respectively^54^ (Fig. 3A, B and Supplemental Table 4). *Gata4* was also noted among the transcripts that defined the VSMC2 subcluster and whose expression was highest in the aortic root, progressively diminishing along the proximal-to-distal axis in the ascending aorta (Fig. 3C). Considering previous work highlighting how cell lineage modulates the effect of LDS-causing mutations^13,55–57^, we explored the relationship between the VSMC2 and VSMC1 subclusters to the secondary heart field (SHF)- and cardiac neural crest (CNC)-lineage of origin (Supplemental Fig. 3). We found that VSMCs lineage-traced with a fluorescent reporter identifying CNC-derived cells were over-represented in the VSMC1 subcluster (Supplemental Fig. 3A). However, re-analysis of a previously published dataset of SHF- and CNC-traced VSMCs (Supplemental Table 5) showed that VSMC1 and VSMC2 were not defined by lineage of origin, with VSMCs of both lineages found in either VSMC sub-cluster^58^ (Supplemental Fig. 3B). Nevertheless, as would be expected based on the known proximal-to-distal distribution of SHF- and CNC-derived VSMCs, there was overlap between VSMC2-defining and SHF-enriched transcripts (Supplemental Fig. 3B, C and Supplemental Table 4 and 5). To assess if the VSMC substructure identified in murine models was relevant in the context of human aortic disease, we also re-analyzed a recently published scRNAseq dataset of aortic tissue from LDS patients and donor aortas in which the ascending aorta and aortic root were separately sequenced (Fig. 3D and Supplemental Fig. 4)^59^. Subpopulations of VSMCs expressing cluster-defining transcripts analogous to those found in VSMC1 and VSMC2 in mouse aortas could be identified in the human dataset (Fig. 3D and Supplemental Table 6). Although both VSMC1 and VSMC2 were present in human aortic root and ascending aorta, GATA4 expression was highest in the VSMC2 cluster from the aortic root, with no detectable expression in the ascending aorta (Fig. 3D).

### *Gata4*-expressing VSMC2 are intrinsically “poised” towards a less-differentiated, maladaptive proinflammatory transcriptional signature.

To examine the biological features of VSMC1 and VSMC2, and whether they were recapitulated in both murine and patient-derived LDS VSMCs, we used the Coordinated Gene Activity in Pattern Sets (CoGAPS) algorithm to identify latent patterns of coordinated gene expression in the *Tgfbr1*^*M318R*/+^ VSMC mouse dataset^60,61^. Two patterns, transcriptional patterns 4 and 5, were found to be enriched in the VSMC2 and VSMC1 subclusters, respectively, in the *Tgfbr1*^*M318R*/+^ VSMC mouse dataset (Fig. 3E, G, Supplemental Table 4). These same patterns were then projected onto the scRNAseq data of VSMCs from the aorta of LDS patients using ProjectR^62^, revealing a similar enrichment of pattern 4 in VSMC2 and pattern 5 in VSMC1 (Fig. 3E–H, Supplemental Table 4).

As previously observed for transcripts upregulated in *Tgfbr1*^*M318R*/+^ LDS VSMCs, Pattern 4-associated transcripts were enriched for transcriptional targets of GATA family members (ENCODE^28^ and ChEA dataset, analyzed with EnrichR^29–32^, Fig. 3I). Differential gene set enrichment analysis using ClueGO^27^ to compare cluster-defining transcripts for VSMC1 and VSMC2 also showed that, in both mouse and human datasets, VSMC2-defining transcripts were enriched for pathways involved in inflammation, senescence, and cellular stress (Fig. 3J and Supplemental Table 7 and Table 8). In contrast, VSMC1 expressed higher levels of transcripts related to extracellular matrix-receptor interactions and contractile function (Fig. 3J, Supplemental Fig. 4 and Supplemental Table 7 and Table 8). Network visualization of molecular signatures database (MSigDB) VSMC2-enriched pathways shared by both mouse and human samples (probed with EnrichR^30–32,63,64^) (Supplemental Fig. 5A), and biological terms with shared ClueGO grouping (Fig. 3J and Supplemental Table 7 and Table 8), highlighted the biological connections between these pathways and genes over-expressed in VSMC2 relative to VSMC1 (i.e. *Cxcl1*^65–68^, *Irf1*^69–71^ *Thbs1*^72^, *Gata4*^73^) (Supplemental Fig. 5B). Overall, in both mouse and human samples, the transcriptional profile of VSMC2 relative to VSMC1 resembled that of less-differentiated VSMCs and included lower expression of *Myh11*, *Cnn1*, and *Tet2*, and higher expression of transcripts associated with non-contractile VSMC phenotypes, including *Klf4*, *Olfm2*, *Sox9*, *Tcf21*, *Malat1*, *Twist1*, and *Dcn*^74–79^.

### *Gata4* is upregulated in the aortic root of *Tgfbr1*^*M318R*/+^ LDS mice.

**Figure 4.**
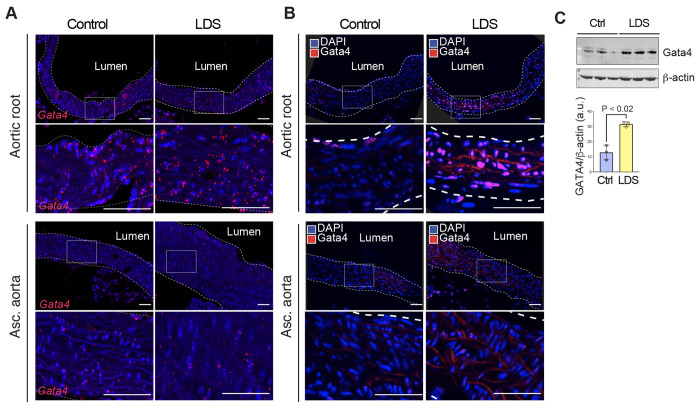
Gata4 mRNA and protein are upregulated in the aortic root of LDS mice. (A) Representative images of RNA in situ hybridization for *Gata4* in the aortic root and ascending aorta of control and LDS (*Tgfbr1*^*M318R*/+^) mice. Insets identify the location shown at higher magnification in the subsequent panel. Scale bars 50 and 200 microns, respectively. (B) Representative images of immunofluorescence for GATA4in the aortic root and ascending aorta of control and LDS mice. Insets identify the location shown at higher magnification in the subsequent panel. Scale bars 50 and 200 microns, respectively. (C) Immunoblot for Gata4 expression relative to ß-actin in aortic root lysates of control (n=3) and LDS mice (n=3), and related quantification of immunoblot, P-value refers to two-tailed Student’s t-test.

**Figure 5.**
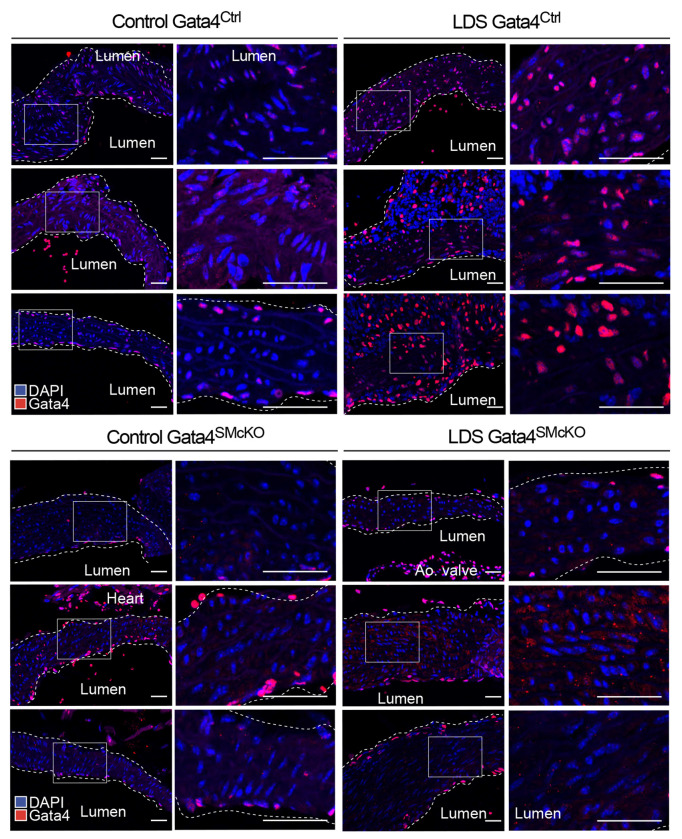
Gata4 protein is upregulated in LDS aortic root of Gata4^Ctrl^ and effectively ablated in Gata4^SMcKO^ mice. Representative images of immunofluorescence for GATA4 at 16 weeks of age. Three independent biological replicates are shown per genotype abbreviated as follows Control (*Tgfbr1*^+/+^) and LDS (*Tgfbr1*^*M318R*/+^) with (Gata4^SMcKO^) or without (Gata4^Ctrl^) smooth muscle specific deletion of Gata4 Insets identify location shown at higher magnification in subsequent panels. Images were acquired at 20x magnification. Scale bars 50 and 200 microns, respectively.

Based on the analysis described above, and its known role in driving the upregulation of pathways previously involved in aneurysm progression^44,73,80^, Gata4 emerged as a potential molecular determinant of increased risk of dilation of the aortic root in LDS. Although levels of *Gata4* mRNA are intrinsically higher in the aortic root relative to the ascending aorta even in control mice (Fig. 3C), its expression was further upregulated in VSMCs in the LDS aorta, as assessed both by scRNAseq (Supplemental Table 1) and RNA in situ hybridization (Fig. 4A). Given that levels of Gata4 protein are highly regulated at the post-transcriptional level through targeted degradation^73,81,82^, we also examined levels of Gata4 protein in control and LDS aortic samples, and found that protein levels are increased in LDS aortic root, both by immunofluorescence and immunoblot assays (Fig. 4B, C and Fig. 5).

### Postnatal deletion of *Gata4* in smooth muscle cells reduces aortic root dilation in LDS mice in association with reduced levels of *Agtr1a* and other proinflammatory mediators.

**Figure 6.**
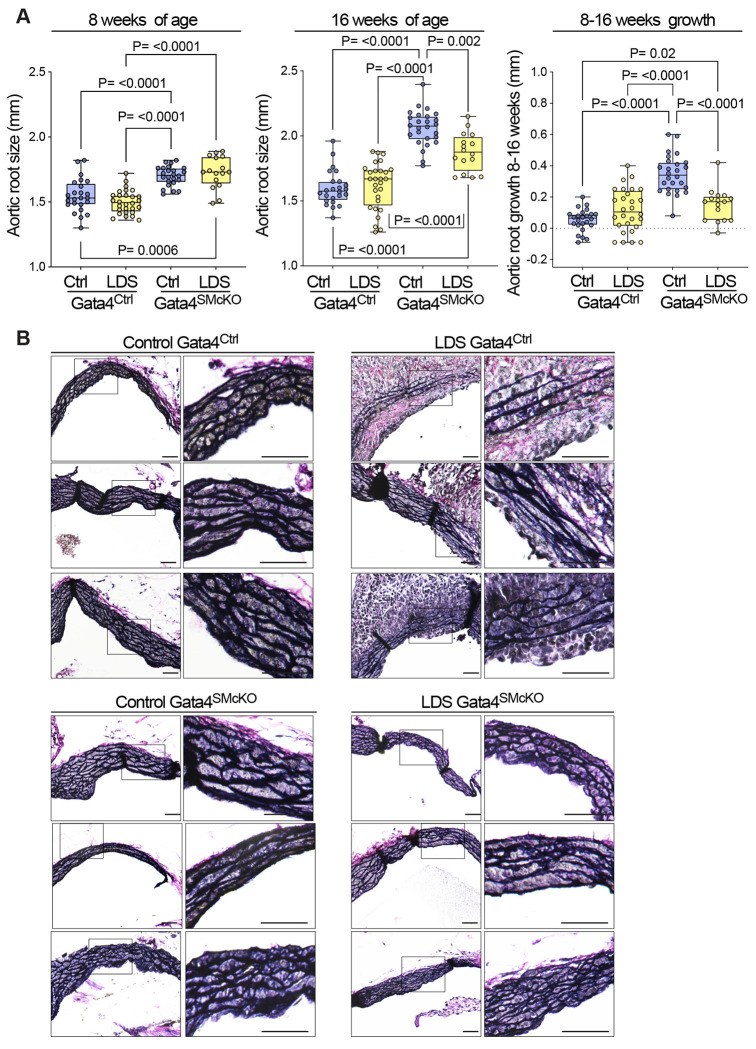
Smooth muscle-specific deletion of Gata4 (Gata4^SMcKO^) reduces aortic root size and growth and improves aortic root media architecture in LDS mice. (A) Aortic root diameter of Ctrl (*Tgfbr1*^+/+^) and LDS (*Tgfbr1*^*M318R*/+^) with (Gata4^SMcKO^) or without (Gata4^Ctrl^) smooth muscle specific deletion of Gata4 as measured by echocardiography at 8 and 16 weeks of age and aortic root growth from 8-16 weeks. P-values refer to Brown-Forsythe AN0VA. (B) Representative VVG-stained aortic root sections from three independent biological replicates per genotype. Insets identify area shown at higher magnification in the subsequent panel. Scale bars 50 and 200 microns, respectively.

To assess whether increased Gata4 levels in aortic root of LDS mouse models promoted dilation in this location, we crossed conditional *Gata4^flox/flox^* mice^83^ to LDS mice also expressing a transgenic, tamoxifen-inducible Cre recombinase under the control of a VSMC specific promoter (*Myh11-Cre*^ER^)^84^, and administered tamoxifen at 6 weeks of age to ablate expression of Gata4 in VSMCs (Fig. 5). VSMC-specific postnatal deletion of Gata4 in LDS mice (*Tgfbr1*^*M318R*/+^; Gata4^SMcKO^) resulted in a reduced rate of aortic root dilation relative to control LDS animals (*Tgfbr1*^*M318R*/+^; Gata4^Ctrl^) (Fig. 6A), and amelioration of aortic root medial architecture relative to control LDS aortas at 16 weeks of age (Fig. 6B). No significant dilation was observed in the ascending aorta of *Tgfbr1*^*M318R*/+^ mice at 16 weeks of age, and Gata4 deletion had no effect on the diameter of this aortic segment (Supplemental Fig. 6). Gata4 deletion in VSMCs also did not associate with changes in blood pressure (Supplemental Fig. 7).

**Figure 7.**
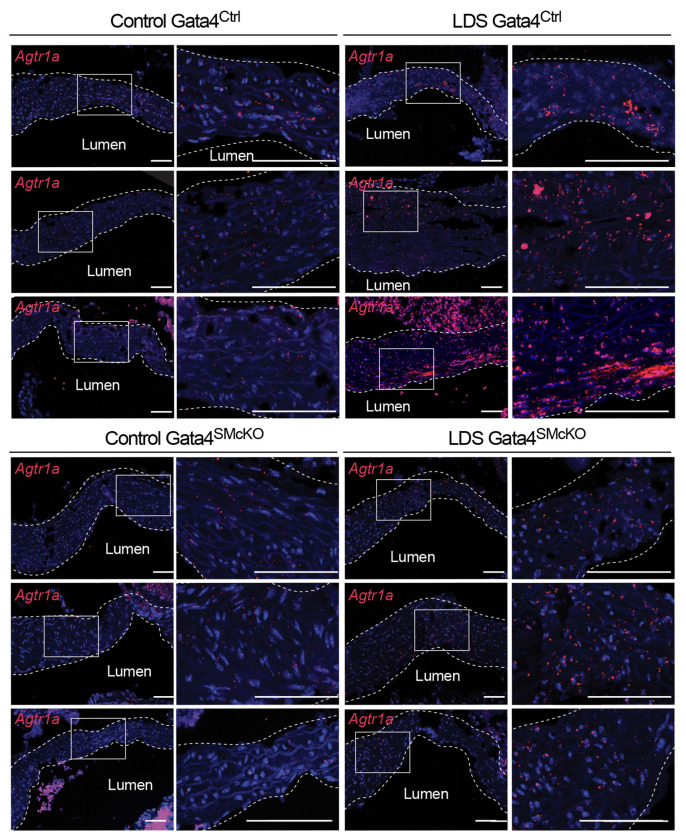
Smooth muscle-specific deletion of Gata4 results in reduced expression of *Agtr1a*. Representative images of RNA in situ hybridization for *Agtr1a* in the aortic root of mice at 16 weeks of age. Three independent biological replicates are shown per genotype abbreviated as follows Control (*Tgfbr1*^+/+^) and LDS (*Tgfbr1*^*M318R*/+^) with (Gata4^SMcKO^) or without (Gata4^Ctrl^) smooth muscle specific deletion of Gata4. Insets identify location shown at higher magnification in subsequent panels. Images were acquired at 20x magnification. Scale bars 50 and 200 microns, respectively.

**Figure 8.**
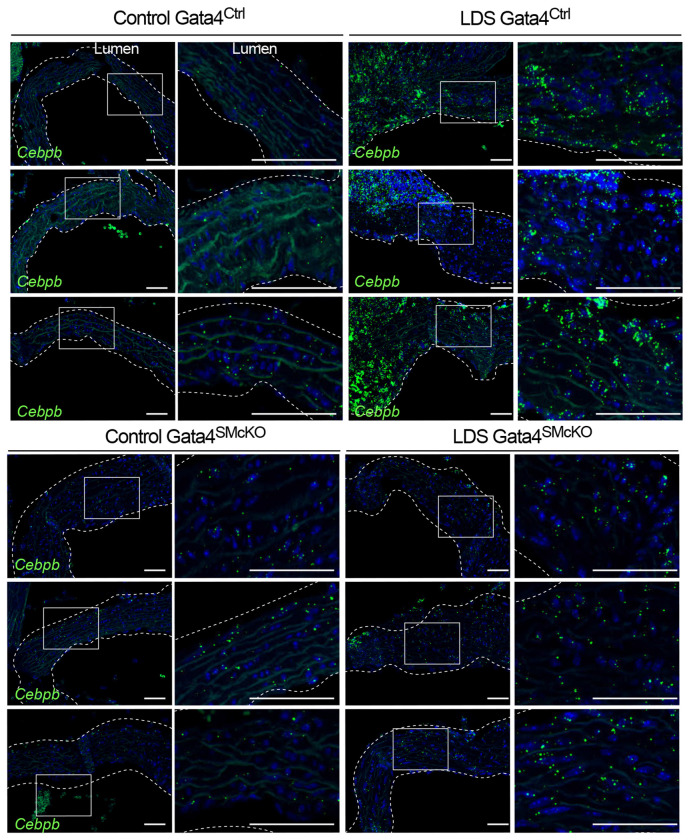
Smooth muscle-specific deletion of Gata4 results in reduced expression of *Cebpb*. Representative images of RNA in situ hybridization for *Cebpb* in the aortic root of mice of indicated genotype at 16 weeks of age. Three independent biological replicates are shown per genotype abbreviated as follows Control (*Tgfbr1*^+/+^) and LDS (*Tgfbr1*^*M318R*/+^) with (Gata4^SMcKO^) or without (Gata4^Ctrl^) smooth muscle specific deletion of Gata4. Insets identify location shown at higher magnification in subsequent panels. Images were acquired at 20x magnification. Scale bars 50 and 200 microns, respectively.

Previous work has shown that Gata4 binds to the *Agtr1a* promoter inducing its expression in heart tissue^44,45^, and that *Agtr1a* is transcriptionally upregulated in the aortic root of LDS mice, resulting in up-regulation of AT1R, which exacerbates LDS vascular pathology^11,13,45^. Accordingly, Gata4 deletion associated with reduced expression of *Agtr1a* in the aortic root of LDS mice (Fig. 7). Similarly, deletion of Gata4 reduced expression of *Cebpd* and *Cebpb* (Fig. 8 and Supplemental Fig. 8), which code for proinflammatory transcription factors regulated by and/or interacting with Gata4 in other contexts^43,46,85,86^, which were highly expressed in VSMC2 relative to VSMC1, and further upregulated in the presence of LDS mutations (Fig. 1, Fig. 2, Supplemental Table 1, Supplemental Table 7).

## Discussion

LDS is a hereditary connective tissue disorder characterized by skeletal, craniofacial, cutaneous, immunological, and vascular manifestations, including a high risk for aggressive arterial aneurysms^4^. It is caused by mutations that impair the signaling output of the TGF-β pathway, leading to defective transcriptional regulation of its target genes^5–9^. Although loss-of-signaling initiates vascular pathology, compensatory upregulation of positive modulators of the pathway results in a “paradoxical” increase in activation of TGF-β signaling mediators (i.e phosphorylated Smad2 and Smad3) and increased expression of target genes in diseased aortic tissue of both LDS patients and mouse models^5,7,10–13^. This secondary upregulation depends, in part, on increased activation of angiotensin II signaling via AT1R, which positively modulates the expression of TGF-β ligands and TGF-β receptors^87^. Whereas upregulation of the TGF-β pathway can have both adaptive and maladaptive consequences depending on disease stage and cellular context^13,54,88–95^, upregulation of AT1R signaling has consistently been shown to be detrimental to vascular health, and both pharmacological (i.e. with angiotensin receptor blockers) and genetic antagonism of this pathway ameliorates vascular pathology in LDS mouse models^87,96–99^.

Even though LDS-causing mutations confer an increased risk of disease across all arterial segments, the aortic root is one of the sites that is particularly susceptible to aneurysm development^14–17^. In this study, we leveraged scRNAseq in conjunction with spatial transcriptomics to investigate the heterogeneity of VSMCs in an LDS mouse model, with the ultimate goal of identifying regional mediators that may drive upregulation of pro-pathogenic signaling in this region. We identify distinct subpopulations of VSMCs characterized by expression patterns that preferentially map to the ascending aorta (VSMC1) and aortic root (VSMC2) in mouse aorta. We also show that the regional vulnerability of the aortic root depends, in part, on higher levels of *Gata4* expression in a subset of VSMCs (VSMC2), which is intrinsically more vulnerable to the effect of an LDS-causing mutation.

Prior to the advent of single-cell analysis tools, which allow precise and unbiased unraveling of cellular identity, the ability to investigate VSMC heterogeneity in the proximal aorta was limited by the availability of experimental approaches to investigate known or expected diversity. In consideration of the mixed embryological origin of the aortic root and distal ascending aorta, earlier work thus focused on understanding how the effect of LDS mutations on VSMCs was modified by the SHF- and CNC lineage of origin. In both mouse models and in iPSCs-derived in vitro models, signaling defects caused by LDS mutations were found to be more pronounced in VSMC derived from SHF (or cardiac mesoderm) progenitors relative to CNC-derived VSMCs^13,57^.

Like SHF-derived VSMCs, *Gata4*-expressing VSMC2 are enriched in the aortic root and are also more vulnerable to the effects of an LDS-causing mutation. They also express a transcriptional signature similar to that of SHF-derived VSMCs (Supplemental Fig. 3). Reciprocally, SHF-derived cells are over-represented in the VSMC2 cluster in our dataset (Supplemental Fig. 3). However, the identity of VSMC2 and VSMC1 is not defined by lineage-of-origin, and SHF- or CNC-derived origin is only an imperfect approximation of the VSMC heterogeneity that can now be assessed via scRNAseq.

Heterogeneity beyond that imposed by lineage-of-origin was also shown by scRNAseq analysis of the aorta of the *Fbn1*^*C1041G*/+^ Marfan syndrome (MFS) mouse model, which revealed the existence of an aneurysm-specific population of transcriptionally modified smooth muscle cells (modSMCs) at a later stage of aneurysmal disease, and which could emerge from modulation of both SHF- and non-SHF (presumably CNC)-derived progenitors^58,100^. These cells, which could also be identified in the aneurysmal tissue derived from the aortic root of MFS patients, showed a transcriptional signature marked by a gradual upregulation of extracellular matrix genes and downregulation of VSMC contractile genes^58,100^. We were not able to identify this population of modSMCs in the aorta of *Tgfbr1*^*M318R*/+^ LDS mouse models, even though it was shown to exist in the aorta of LDS patients^62^.

Similar to the early effect of Smad3-inactivation, the *Tgfbr1*^*M318R*/+^ LDS mutation caused broad downregulation of gene programs required for extracellular matrix homeostasis and those favoring a differentiated VSMC phenotype^54^ (Fig. 1); conversely, proinflammatory transcriptional repertoires, with an enrichment in pathways related to cell stress, was observed among upregulated transcripts. This latter profile likely represents a response to the initial insult caused by decreased expression of extracellular matrix components whose expression requires TGF-β/Smad activity^98^.

We also noted downregulation of several components of the lysosome, whose function is required for cellular homeostasis and degradation of protein targets via selective autophagy^33,73,101,102^ (Fig. 1). Gata4 levels are regulated via p62-mediated selective autophagy^73^ and by mechanosensitive proteasome-mediated degradation^82,103^. The aortic root would be especially vulnerable to a defect in either of these processes given increased baseline levels of *Gata4* mRNA expression in VSMC2. Increased levels of Gata4 may contribute to vascular pathogenesis by several potential mechanisms. In other cellular contexts, Gata4 has been shown to promote induction of the pro-inflammatory senescence-associated secretory phenotype (SASP) as well as transcription of the lncRNA *Malat1*, which promotes aneurysm development in other mouse models^78^. Gata4 is also a negative regulator of contractile gene expression in Sertoli and Leydig cells^104^. Additionally, Gata4 binds the promoter and activates the expression of *Agtr1a*^44^, which is known to drive pro-pathogenic signaling in LDS aorta^45^. Accordingly, we find that Gata4 deletion downregulates expression of *Agtr1a* in the aortic media of LDS mouse models (Fig. 7).

Re-analysis of a scRNAseq dataset of human aortic samples from LDS patients, which included both the aortic root and the ascending aorta, shows that a population of *Gata4*-expressing VSMC similar to that found in mice can also be identified in LDS patients. Additionally, patterns of coordinated gene expression identifying VSMC1 and VSMC2, which were learned from the scRNAseq analysis of mouse aorta, could be projected onto the human dataset, suggesting that these two subsets of VSMCs are conserved across species and that the existence of a *Gata4*-expressing VSMC2 population may underlie increased risk in the aortic root of LDS patients as well. Assessing the effects of Gata4 deletion at additional postnatal timepoints will be important to understand the consequences of increased Gata4 and its downstream targets during later stages of disease. Although direct targeting of Gata4 for therapeutic purposes is unfeasible given its critical role in the regulation of numerous biological processes in non-vascular tissues^105–109^, this work highlights how the investigation of factors that increase or decrease the regional risk of aneurysm may lead to a better understanding of adaptive and maladaptive pathways activated in response to a given aneurysm-causing mutations. This knowledge may be leveraged to develop therapeutic strategies that target the vulnerabilities of specific arterial segments.

## Methods

### Animal Experiments

#### Study approval

Animal experiments were conducted according to protocols approved by the Johns Hopkins University School of Medicine Animal Care and Use Committee.

#### Mouse models

All mice were maintained in an animal facility with unlimited access to standard chow and water unless otherwise described. *Tgfbr1*^+/+^ and *Tgfbr1*^*M318R*/+ 11^(The Jackson Laboratory, strain #036511) mice, some bearing the *EGFP-L10a*^110^ (The Jackson Laboratory, strain #024750) conditional tracer allele and a CNC-specific CRE recombinase expressed under the control of Wnt2 promoter^111^ (The Jackson Laboratory, strain #003829) were used for scRNAseq as described below. All mice were maintained on a 129-background strain (Taconic, 129SVE). *Tgfbr1*^+/+^ and *Tgfbr1*^*M318R*/+^ mice were bred to *Gata4^flox/flox^* ^83^(The Jackson Laboratory, strain #008194) and mice carrying the *Myh11-Cre^ER^* transgene^84^ (The Jackson Laboratory, strain #019079). *Myh11-Cre^ER^* is integrated on the Y chromosome therefore only male mice were used for this set of experiments. *Tgfbr1*^+/+^ and *Tgfbr1*^*M318R*/+^ bearing *Gata4^flox/flox^* and *Myh11-Cre^ER^* are referred to as Gata4^SMcKO^. *Tgfbr1*^+/+^ and *Tgfbr1*^*M318R*/+^ bearing *Gata4*^+/+^ with or without *Myh11-Cre^ER^* or *Gata4^flox/flox^* or *Gata4*^*flox*/+^ without *Myh11-Cre^ER^* are referred to as Gata4^Ctrl^. All Gata4^SMcKO^ and Gata4^Ctrl^ mice were injected with 2 mg/day of tamoxifen (Millipore Sigma, T5648) starting at 6 weeks of age for 5 consecutive days. Mice were genotyped by PCR using primer sequences described in the original references for these models. Serial echocardiography was performed using the Visual Sonics Vivo 2100 machine and a 30 MHz probe. As there is some variability in the onset of aortic dilation in *Tgfbr1*^*M318R*/+^ mice, and starting aortic size will affect final measurements, aortic root diameter of 1.9 mm and above at baseline (8 weeks of age) was defined *a priori* as an exclusion criterion.

### Molecular validation techniques

#### Aortic Sample Preparation

All mice were euthanized by halothane inhalation at a 4% concentration, 0.2 ml per liter of container volume (Millipore Sigma, H0150000). As we described previously^11,54^, the heart and thoracic aorta were dissected en bloc and fixed in 4% paraformaldehyde (Electron Microscopy Sciences, 15710) in PBS at 4°C overnight. Samples were subsequently incubated in 70% ethanol at 4°C overnight prior to embedding in paraffin. Paraffin-embedded tissues were cut into 5 micron sections to expose a longitudinal section of the thoracic aorta. Sections were then stained with Verhoeff-van Gieson (StatLab, STVGI) to visualize elastic fiber morphology or to assess protein and RNA abundance by immunofluorescence or fluorescence in situ hybridization.

#### Immunofluorescence

Immunofluorescence was performed following a protocol adapted from Cell Signaling Technology (CST) for formaldehyde-fixed tissues as previously described in detail^45^, using a rabbit monoclonal antibody for GATA4 (Cell Signaling Technology, CST36966) and a donkey anti-rabbit secondary antibody Alexa Fluor 555 (ThermoFisher, A32794). Images were taken using a Zeiss LSM880 Airyscan FAST confocal microscope at 20× magnification and are presented as maximal intensity projection.

#### RNAscope Fluorescence in situ hybridization

RNA in situ hybridization was performed using the RNAscope Multiplex Fluorescent Reagent Kit v2 Assay (ACD Biosciences, 323100) according to the manufacturer’s protocol with the following probes *Mm-Gata4* (417881), *Mm-Agtr1a* (481161), *Mm-Cebpd* (556661), *Mm-Cebpb* (547471). Images were taken using a Zeiss LSM880 Airyscan FAST confocal microscope at 20× magnification and are presented as maximal intensity projection.

#### Immunoblotting

Aortic root tissue was flash-frozen immediately upon dissection and stored at −80°C until protein extraction. Protein was extracted using Full Moon Lysis Buffer (Full Moon Biosystems, EXB1000) with added phosphatase and protease inhibitors (MilliporeSigma, 11836170001 and 4906845001) and Full Moon lysis beads (Full Moon Biosystems, LB020) using an MP Biomedicals FastPrep 24 5G automatic bead homogenizer. After homogenization, the cell debris was pelleted, and the supernatant was collected. Immunoblot was performed as previously described in detail^54^, using a rabbit monoclonal antibody for Gata4 (Cell Signaling Technology, 36966) and a mouse monoclonal antibody for ß-Actin. (Cell Signaling Technology, 8H10D10).

### Transcriptomic Analyses

#### Single Cell RNA sequencing and analysis

Single cell RNA sequencing was performed as we previously described^112^. Single cell suspensions from each mouse were processed separately using the 10x Genomics 3’ v3 platform and sequenced on an Illumina NovaSeq. A total of 30,704 aortic cells were sequenced from six female mice. The raw data was processed, aligned to the mouse genome (mm10), and aggregated using 10x Genomics Cell Ranger V6^113^. The data were then filtered using the Seurat V5 package^112^ based on the following criteria: >1000 transcripts detected per cell but <5000, >1500 total molecules detected per cell but <25000, and <20% mitochondrial transcripts per cell. Filtering reduced this dataset from 30,704 aortic cells to 24,971 cells for further analysis. The data was then normalized using the function SCTransform v2. As samples were prepared on multiple days, the data was integrated across batches using reciprocal principal component analysis (RPCAIntegration). Principal component analysis and uniform manifold approximation and projection (UMAP) were performed followed by the FindNeighbors and FindClusters functions. We opted to cluster at a low resolution (0.25) to differentiate aortic cell types and to identify only major subpopulations of smooth muscle cells that vary by a large number of differentially expressed genes. FindMarkers was used to identify cluster-defining transcripts and differentially expression genes between control and diseased cell populations based on a Wilcoxon rank sum test.

#### Re-analysis of human aortic cells from Pedroza et al., 2023

For re-analysis of the ascending aorta and aortic root samples from a recently published scRNAseq dataset of the donor and LDS patient aortas^59^ we used the following criteria: > 1000 transcripts detected per cell but< 6000, > 1500 total molecules detected per cell < 30000, and < 20% mitochondrial transcripts per cell. This reduces this dataset from 58,947 aortic cells to 43,349 for further analysis. We analyzed this dataset as described above with the FindClusters resolution parameter set to 0.15.

#### CoGAPS and ProjectR

CoGAPS^60,61^ (v3.22), an R package that utilizes non-negative matrix factorization to uncover latent patterns of coordinated gene expression representative of shared biological functions, was used to identify transcriptional patterns associated with VSMC subpopulations, with the npatterns parameter set to 8, in scRNAseq analysis of murine aortas. ProjectR^62^ (v1.2), an R package that enables integration and analysis of multiple scRNAseq data sets by identifying transcriptional patterns shared among datasets, was used to project these patterns into scRNAseq analysis of the human aortic root and ascending aorta.

#### Gene over-representation analyses

ClueGO^27^ was used for gene over-representation analysis and visualization of enriched functional terms for transcripts globally dysregulated in all VSMCs as well as VSMC subsets. Transcripts were filtered based on an adjusted P-value less than 0.05 and an average absolute Log2 fold change of 0.25 or greater, as well as detection in at least 20 percent of either control or LDS VSMCs. The resulting list of 502 downregulated and 200 upregulated genes was compared against five gene ontology databases (MSigDB Hallmark, KEGG, WikiPathways, Bioplanet, and Reactome). The list of transcripts and ClueGO log files are provided in supplemental material. Differentially expressed gene lists were also analyzed using the online gene list enrichment analysis tool EnrichR^30–32^ (https://maayanlab.cloud/Enrichr/) for pathways using the Molecular Signatures Database (MSigDB)^63,64^ and for transcription factors target enrichment using the ENCODE^28^ and ChEA^29^ databases.

#### Multiplexed Error-Robust Fluorescence in situ Hybridization (MERFISH) Spatial Transcriptomics

MERFISH spatial transcriptomics using a custom panel was performed on 5-micron Formalin-Fixed Paraffin-Embedded (FFPE) sections of control and LDS aortas according to manufacturer’s protocols (MERSCOPE FFPE Tissue Sample Preparation User Guide_Rev B, Vizgen). Slides were processed and imaged on a MERSCOPE instrument platform according to the manufacturer’s protocols (MERSCOPE Instrument User Guide Rev G, Vizgen). The raw images were processed by the instrument software to generate a matrix of spatial genomics measurements and associated image files that were analyzed using the MERSCOPE visualizer software.

### Statistics

GraphPad Prism 10.0 was used for data visualization and statistical analysis. Data tested for normality using the Shapiro-Wilk test and upon verification of normal distribution, analyzed using the Brown-Forsythe ANOVA test. For echocardiographic and blood pressure measurements, data are presented as a box and whisker plot with the whiskers indicating the maximum and minimum values and a horizontal bar indicating the median. All individual data points are shown as dots. Figures indicating statistical significance include the statistical tests used in the figure caption.

## Data Availability

All single-cell RNA sequencing data, both raw fastq files and aggregated matrixes, will be available in the gene expression omnibus (GEO) repository under accession number GSE267204. MERFISH spatial transcriptomics data is available upon request.
